# The physiological stress response of juvenile nurse sharks (*Ginglymostoma cirratum*) to catch-and-release recreational angling

**DOI:** 10.1371/journal.pone.0316838

**Published:** 2025-01-03

**Authors:** Katherine C. Giesy, Jacob Jerome, Julia Wester, Evan D’Alessandro, M. Danielle McDonald, Catherine Macdonald

**Affiliations:** 1 University of Miami Rosenstiel School of Marine, Atmospheric, and Earth Science, Miami, Florida, United States of America; 2 Field School, Coconut Grove, Florida, United States of America; University of Iceland, ICELAND

## Abstract

Nurse sharks (*Ginglymostoma cirratum*), especially juveniles, are often encountered by near-shore and shore-based recreational anglers and are suggested to exhibit minimal behavioral and physiological responses to capture, largely based on studies of adults using commercial or scientific fishing methods. To quantify the sub-lethal effects of recreational angling on juvenile nurse sharks, 27 individuals (across 31 angling events) were caught using hook-and-line fishing methods. Over a 30-min period, 4 blood samples were taken with variable time intervals between sampling (*i*.*e*., randomized ordering of an interval of 5, 10, and 15 min between each sampling event). Lactate increased by 611% (6.7 ± 2.17 mmol/L) on average over the 30-min fight, and significant relationships were identified between lactate and blood draw number, fight time, and temperature, with large effect sizes. Significant relationships were also detected between blood draw number, glucose, and hematocrit, while osmolality was only affected by fishing site. These results suggest juvenile nurse sharks may exhibit a greater physiological stress response when exposed to recreational angling than adults captured with other fishing methods.

## Introduction

Shark populations have been in decline worldwide due to the rapid growth of global fishing capacity coupled with high bycatch rates [1, 2]. In addition, sharks generally have life history characteristics (*e*.*g*., low fecundity, late age at maturity, and relatively slow growth rates) that constrain reproductive capacity and render them intrinsically sensitive to fishing mortality [3, 4]. Despite these concerns, recreational angling is a popular leisure activity worldwide [5] with recreational landings of sharks in the United States exceeding commercial landings in recent years [6, 7], furthering the need to better understand the impacts of non-lethal recreational fisheries interactions on shark populations.

Although the practice of catch-and-release fishing is advocated in recreational fisheries, questions remain about levels of post-release mortality [8, 9] as well as more cryptic sub-lethal effects on growth and fitness due to stress, injuries, and increased susceptibility to predation [9–12]. While some studies have examined the physiological stress caused by catch-and-release fishing on sharks using other fishing techniques (*e*.*g*., longlining or drumlining) [13–15], there have been comparatively fewer studies evaluating the physiological stress response of sharks to common recreational angling gears or methods [16, 17].

Unlike large-scale commercial fisheries, which typically take place at a greater distance from shore, recreational anglers often access coastal environments that are critical habitat for many fish species at multiple life stages and can be especially important for juveniles [11, 18]. While shallow waters may offer young teleost fishes and sharks protection from natural predators or access to coastal resources, proximity to land increases the probability of recreational fishing interactions and exposes them to capture stress during a critical life stage [19–21]. Additionally, species with nearshore distributions and strong site fidelity, like the nurse shark (*Ginglymostoma cirratum*), are more accessible to recreational anglers, including the probability of repeated captures, and in turn, are potentially more susceptible to angling-related impacts. Nurse sharks are a coastal species found in the eastern Pacific and western Atlantic Oceans [22] with neonates and juveniles remaining close to shore in seagrass flats and mangrove islands [23]. Tagging studies have revealed a high degree of site fidelity in adults, including long-term mating site fidelity [24], and particularly limited movement patterns compared to other shark species [22–23, 25]. The Everglades National Park Creel survey reported that between 1972 and 2002, nurse sharks were the second most frequently caught species from sport fishing trips [26]. Although nurse sharks are common in South Florida and are often encountered in recreational fisheries, and their population there is believed to be increasing, the western Atlantic population overall is listed as “Vulnerable” by the IUCN with a decreasing population trend [27] due to ongoing fishing pressures, habitat degradation, and the impacts of climate change [27, 28].

As a resident, largely non-migratory species, the nurse shark is especially susceptible to exposure to repeated capture stress in frequently fished environments. A study by Hammerschlag et al. [29] found nurse sharks in Biscayne Bay did not avoid urbanized areas, even during periods of higher human activity. Furthermore, Whitney et al. [30] reported nurse sharks have the lowest mass and temperature-adjusted metabolic rate known for any shark species, and suggested they may incur a much higher relative metabolic cost of activity in switching from rest to movement compared to more active elasmobranch species. Perhaps as a result of these characteristics, nurse sharks exhibit a relatively passive “fight” during capture compared to other species [31, 32]. However, these studies captured adults and used commercial or scientific fishing methods that have relatively long standardized soak times. Therefore, they lack key data on how juveniles respond to capture and the behavioral and physiological responses that this species exhibits as a result of recreational angling methods (*i*.*e*., a continuous active fight). With a relatively high cost of movement and low metabolic rate, nurse sharks are energetically suited for, and perhaps dependent on, a minimally active lifestyle [30] and may experience negative physiological consequences if forced to actively ‘fight’ on a line. Identification of these angling-related consequences and their impact is essential for the development of best management and conservation practices for this species as they are often captured by shore-based recreational anglers [26] and they are commonly perceived as a robust shark species by both scientists [13] and recreational anglers in South Florida [33].

The purpose of this study was to collect data on the physiological stress response of juvenile nurse sharks (*Ginglymostoma cirratum*) to catch-and-release recreational angling and the potential influence of biotic and abiotic factors in shaping that response. Specifically, we sought to measure the physiological status of juvenile nurse sharks at multiple points in time during an active fight on hook and line. To assess these impacts, lactate, glucose, hematocrit and osmolality were measured, and the relationship between these parameters and fight time, shark size, and water temperature were evaluated. This is the first study to examine the physiological disturbance caused by recreational angling in nurse sharks and therefore provides information on a data limited species. Understanding these physiological impacts could have applications for species-specific management of recreational fisheries and better support conservation initiatives for nurse sharks.

## Materials and methods

### Study site, fishing and capture methods

Sampling was conducted in Northern Biscayne Bay near Miami, Florida from November 2019 to October 2020 along shorelines with similar depths ranging from 3 to 9 feet. Biscayne Bay is a large, shallow marine estuary encompassing coral patch reefs, seagrass beds and mangrove shoreline. The Bay serves a significant ecological role, supporting substantial biodiversity [34, 35]. Biscayne Bay has experienced rapid urbanization due to human population growth over the course of the 20th century as it borders Miami-Dade County, Florida, a major U.S. county with a current population of approximately 2.67 million people [36]. Urban land use change and anthropogenic pressure has resulted in habitat degradation, eutrophication, overfishing and localized die-offs of seagrasses, mangroves, and coral reefs [37].

Sharks were captured via hook and line using 8-foot recreational rods equipped with 50 lb. test monofilament, a stainless steel leader, and size 13/0 barbed circle hooks to reduce the risk of foul hooking (*i*.*e*., hooking in any part of the body other than the jaw) and increase the probability of successful landing. The time was recorded from the first sign a shark was detected on the line until the initial blood sample was taken (“time 0”), based on the methods of Hoffmayer and Parsons [38]. Once hooked, sharks were retrieved as quickly as possible (within 3 min) to allow for the best estimate of pre-disturbance blood parameters [39]. After the initial blood sample, each shark remained on the hook-and-line and was returned to the water where it was angled for the additional time between blood samples. Between blood draws the sharks were continuously engaging in active swimming against the line and not allowed to rest. Over a period of 30 min, 4 small blood samples (2 ml each) were drawn from the caudal vein of each animal. After each blood sample, the shark was returned to the water and the fight continued with each subsequent blood sample taken with variable fight time intervals between sampling (*i*.*e*., randomized ordering of an interval of 5, 10, and 15 min between each sampling event to control for the influence of shark handling and the blood draw procedure; Table 1). During the final landing, the sharks were also rapidly measured (pre-caudal length, fork length, total length, and girth), sexed (visual survey of cloaca), and tagged with small, plastic Floy-brand spaghetti tags (individuals < 1 m) or NOAA cooperative tags (individuals > 1 m; S1 Dataset). Lastly, small fin clip samples were collected to be used in future genetic studies, and surface water temperature was recorded.

**Table 1:** Example of different blood sampling time interval schedules for juvenile nurse sharks.

Shark	Fight Time Interval (minutes)			
	Blood Draw 1	Blood Draw 2	Blood Draw 3	Blood Draw 4
1	0	15	10	5
2	0	10	5	15
3	0	5	10	15
4	0	15	5	10
5	0	10	15	5
6	0	5	15	10

Animal research was approved by the University of Miami IACUC Review Board (IACUC # 19–167). Field research was permitted under Florida Fish and Conservation Commission (FWC) (Permit # 19-SAL-1798).

### Blood draw and processing

At each time interval, individuals were retrieved from the water and whole blood samples (<2 ml) were drawn with 18-gauge 3-inch needles within 1 min of landing the shark via caudal venipuncture. Lactate and glucose were immediately measured by adding approximately 10 μl of blood each to a glucose meter (Accu-Chek glucose meter; Roche Diagnostics, Basel, Switzerland) and to a lactate meter (Lactate Pro 2 LT-1730 portable lactate analyzer; Arkray Inc., Kyoto, Japan). The remainder of the blood was then added to blood collection tubes spray-coated with lithium heparin (BD Vacutainer® Heparin Tubes) and stored on ice until returning to the lab. Hematocrit was immediately determined upon returning to the lab through centrifugation (13,000 g x 5 min) of plain capillary tubes and visual measurement of the packed red blood cell volume (% Hct). The remainder of the heparinized blood was transferred into vials and spun via centrifugation (13,000 g x 5 min) for plasma extraction. Vials containing plasma were frozen at -20°C for later lab-based analysis using a Westcor Vapor Pressure Osmometer to measure osmolality.

### Statistical analyses

Linear mixed models (LMM) were used to explore how total fight time (the total amount of time a shark has been on the line) and fight time (the amount of time the shark fought on the line between blood draws, *i*.*e*., 5, 10, or 15 min) relate to physiological parameters (lactate, glucose, hematocrit and osmolality). Total length, sex, water temperature, and fishing site were also included in the analysis as covariates. To control for the influence of shark handling and the blood draw procedure, the order of the fight time intervals (5, 10, and 15 min) varied randomly. Additionally, two variations of the model were used for each physiological parameter. The first model for each physiological parameter explored total fight time while the second model included blood draw number as a variable to investigate the potential influence of the blood draw procedure on the model output while also investigating relationships between physiological parameters and fight time. A separate LMM was used to determine the relationship between initial levels of each physiological parameter and the time it took to obtain the initial blood sample. For all the models, a random intercept (shark ID number) was included to account for between-subject variation. The sum of squared residuals was calculated for each model. Relative effect sizes were computed following Cohen [40] with *d* = 0.2 representing a small effect size, *d* = 0.5 a medium effect size, and *d* = 0.8 a large effect size. Individual models were run using the “lme4” and “EMAtools” packages in R version 3.6.3 (R Core Team, 2020) and statistical significance was set *a priori* at p < 0.05 (S2 Dataset).

Based on Giesy [41], the Lactate Pro meter overestimates lactate concentrations, but in a predictable way. Therefore, all lactate concentrations were converted using a calculated adjustment curve equation (y = 1.79x – 0.08). There were no differences in statistical significance for the variables in either model between the original Lactate Pro values and the adjusted lactate concentrations. The Accu-Chek glucose meter has not been validated with laboratory analysis. Therefore, the data presented were not converted to laboratory-based values.

## Results

Between November 2019 and October 2020, a total of 27 nurse sharks (15 females, 12 males), with a total length ranging from 63 to 153 cm (mean ± SD = 101.8 ± 25.0 cm), were caught and sampled (Table 2). Four sharks were recaptured and resampled (31 total angling events) one of which was caught three separate times and another of which was recaptured one day after being caught for the first time. The remaining two sharks were recaptured after 14 days and assumed to be fully recovered; therefore, data from all capture events were pooled for analysis. Additionally, one shark was able to break free from the line before the final blood draw could be performed. This individual had a NOAA cooperative tag and total length was estimated based on previous capture data from seven months prior linked to the tag. It was found that sex had no significant (all *p* > 0.05) effect on the stress response across any physiological parameter, so it was excluded from the models. Water temperature at the time of capture ranged from 21.1 to 33.9°C (mean ± SD = 30.5 ± 3.4°C). Fishing site was found to only be significant for osmolality (all other *p* > 0.05), so it was excluded from the other physiological parameter models.

**Table 2:** Juvenile nurse shark physical characteristics.

Number of			Sex		Total Length (cm)	Girth (cm)
Sharks	Recaptures	Total	M	F	Range	Range
27	4	31	12	15	63–153	16–40
					Average (± SD)	Average (± SD)
					101.77 ± 24.89	27.10 ± 5.70

All initial blood samples in this study were collected within 3 min (mean ± SD = 1.84 ± 1.02 min) with the exception of two of the largest sharks. There were no statistically significant relationships between initial levels of lactate, glucose, hematocrit or osmolality and the time (0.55 to 2.58 min for all other sharks, 4.25 and 5.33 min for the two largest sharks) it took to obtain the initial blood sample (Lactate: *p* = 0.195, Glucose: *p* = 0.297, Hematocrit: *p* = 0.469, Osmolality: *p* = 0.519). The average initial lactate value was 1.22 ± 0.64 mmol/L (Table 3).

**Table 3:** Juvenile nurse shark physiological blood parameter data. A) Minimum and maximum values of each blood parameter collected from all sharks. B) Average of all initial values collected at Time “0” across all sharks. C) Average amount of change in each blood parameter from Time “0” to Time “30” across all sharks.

**A) Value range:**			
Lactate (mmol/L)	Glucose (mmol/L)	Hematocrit (%)	Osmolality (mOsm/kg)
0.7–22.1	1.7–9.6	11.1–26.4	622.0–1042.0
**B) Average initial values (± SD):**			
Lactate (mmol/L)	Glucose (mmol/L)	Hematocrit (%)	Osmolality (mOsm /kg)
1.2 (±0.64)	3.0 (±0.66)	17.5 (±2.66)	891.3 (±106.33)
**C) Average change in (± SD):**			
Lactate (mmol/L)	Glucose (mmol/L)	Hematocrit (%)	Osmolality (mOsm /kg)
6.70 (±2.17)	2.2 (±0.87)	-0.70 (±1.87)	-17.9 (±196.84)

**Fig 1:**
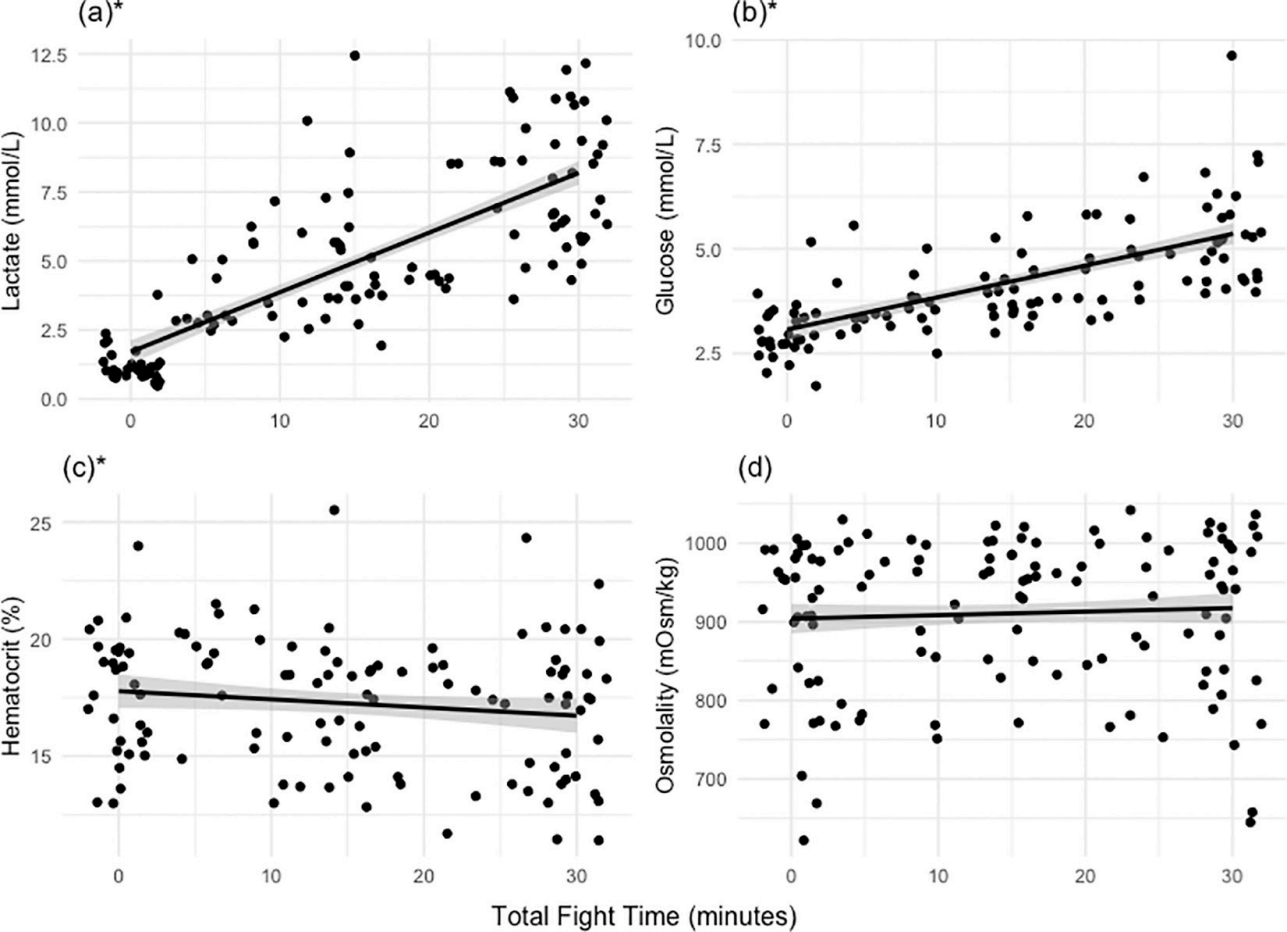
Relationships between total fight time and physiological status of juvenile nurse sharks (*Ginglymostoma cirratum*). Significance is denoted with a (*). Lactate (a), glucose (b), and (c) hematocrit concentrations were measured from whole blood, and osmolality (d) concentrations were measured from plasma. Lines represent the fitted regression of all the sharks’ predicted physiological parameters generated from the Linear Mixed Effects Models. Confidence intervals are also displayed as grey shading.

**Fig 2:**
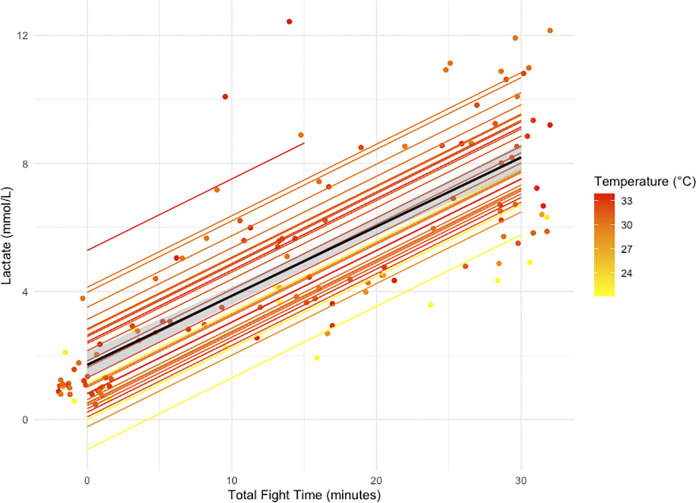
The change in lactate (mmol/L) values across all angling events (n = 31), represented as points, plotted with lines that show the predicted change in lactate (mmol/L) values over 30-min total fight time from the linear mixed effects model outputs. Colored lines represent the fitted regression of predicted lactate values per individual shark color coded according to temperature (°C) and the black line is the overall slope of all the sharks’ predicted lactate values over total fight time.

Lactate increased by an average of 611% (6.7 ± 2.17 mmol/L) over the 30-min fight (Table 3 and Fig 1A). Furthermore, the LMM established a significant linear relationship between lactate, total fight time, and temperature (Table 4 and Fig 2). When blood draw number was added as a variable, model results indicated that blood draw number, fight time, and temperature were all significantly related to lactate (Table 4). Glucose values increased by 72% (2.2 ± 0.87 mmol/L) (Table 3 and Fig 1B), but only significantly changed with blood draw number (Table 4). Hematocrit decreased by 0.7 ± 1.9% on average over the 30-min fight (Table 3 and Fig 1C). Model results indicated increasing total fight time and blood draw number were inversely related to hematocrit and total length was positively related to hematocrit values (Table 4). Osmolality decreased by 1.2% (17.9 ± 196.8 mOsm/kg) over the 30-min fight time (Table 3 and Fig 1D), but fishing location was the only variable to have a significant correlation to osmolality (Table 4).

**Table 4:** Linear mixed effects model outputs (95% confidence interval limits) for the effect of total fight time, fight time, temperature, total length and fishing site on the physiological parameters. Bold denotes statistically significant factors.

	Variable	Estimate (β)	CI	*p*	Effect size (*d*)	Adj. *R*^*2*^
***Lactate***						
	Model not including blood draw number as a variable					0.833
	**Total fight time**	0.22	0.20 – 0.24	**<0.001**	4.96	
	**Temperature**	0.11	0.01 – 0.21	**0.040**	0.80	
	Total length	-0.01	-0.03 – 0.02	0.703	-0.08	
	Model including blood draw number as a variable					0.842
	**Blood draw**	1.95	1.75 – 2.16	**<0.001**	4.99	
	**Fight time**	0.08	0.04 – 0.12	**<0.001**	1.06	
	**Temperature**	0.14	0.02 – 0.26	**0.027**	0.87	
	Total length	-0.02	-0.04 – 0.01	0.299	-0.28	
***Glucose***						
	Model not including blood draw number as a variable					0.863
	**Total fight time**	0.07	0.06 – 0.08	**<0.001**	3.81	
	Temperature	0.02	-0.04 – 0.09	0.416	0.31	
	Total length	-0.01	-0.02 – 0.01	0.316	-0.23	
	Model including blood draw number as a variable					0.841
	**Blood draw**	0.74	0.59 – 0.89	**<0.001**	2.90	
	Fight time	0.03	-0.01 – 0.06	0.065	0.55	
	Temperature	0.04	-0.03 – 0.10	0.289	0.41	
	Total length	-0.01	-0.03 – 0.01	0.234	-0.35	
***Hematocrit***						
	Model not including blood draw number as a variable					0.453
	**Total fight time**	-0.03	-0.05 – -0.01	**0.011**	-0.56	
	Temperature	0.03	-0.13 – 0.19	0.706	0.14	
	**Total length**	0.04	0.00 – 0.08	**0.044**	0.81	
	Model including blood draw number as a variable					0.436
	**Blood draw**	-0.46	-0.79 – -0.13	**0.007**	-0.74	
	Fight time	-0.04	-0.10 – 0.03	0.293	-0.28	
	Temperature	0.01	-0.15 – 0.18	0.866	0.07	
	Total length	0.04	-0.00 – 0.08	0.052	0.78	
***Osmolality***						
	Model not including blood draw number as a variable					0.469
	Total fight time	0.60	-0.44–1.64	0.254	0.24	
	Temperature	-2.33	-5.66–1.0	0.163	-0.54	
	Total length	0.09	-0.79–1.65	0.486	0.05	
	**Fishing site**	143.43	96.35–190.51	**<0.001**	2.36	
	Model including blood draw number as a variable					0.474
	Blood draw	-8.46	-24.99–8.06	0.296	-0.27	
	Fight time	1.76	-1.55–5.06	0.305	0.28	
	Temperature	-2.42	-5.73–0.89	0.428	-0.57	
	Total length	-0.04	-0.85–0.77	0.637	-0.03	
	**Fishing site**	141.34	94.54–188.14	**<0.001**	2.33	

## Discussion

The stress from a capture event elicits a complex physiological response in fish. Although this physiological response has been relatively well-studied for some elasmobranchs [15, 42–44], little research has been done to understand this response in nurse sharks, and what is known focuses on adults and scientific fishing methods [31, 32]. Their strong site fidelity and low metabolic rates make them particularly susceptible to repeated captures at recreational fishing sites and chronic exposure to localized stressors [22, 23]. Indeed, in this study, a total of four angling events were of recaptured sharks, with one shark caught three separate times and one caught only one day after it was first captured. It has been well-documented in previous studies that other species of elasmobranchs use shallow, subtidal zones as refuges from predators, and it is likely that juvenile nurse sharks are using these fishing sites around shallow, highly impacted urban areas (*e*.*g*., areas near marinas and sea walls) for protection from predation [23, 45]. Recaptures in this study suggest they may be site attached with small home ranges, which is supported by existing literature on other species [22, 23, 25]. For example, juvenile lemon sharks (*Negaprion brevirostris*) use subtidal mangrove habitat to decrease the likelihood of encounters with predators even under conditions of decreased habitat quality [46, 47]. Although nurse sharks appear to be generally more resilient to capture stress than many other species [13, 14], their strong site fidelity increases the risk of repeated captures and subsequent physiological responses. This potential chronic stress has been shown in other species to suppress growth, reduce reproductive success, and limit foraging energy, and can lead to increased predation risk and reduced survival [48, 49].

The results of the LMM for lactate revealed a significant linear relationship with fight time, although this and other physiological studies have been unable to distinguish between the stress response to an initial hooking event and the response to the “fight”. The relationship between fight time and lactate is reflected in existing literature including for juvenile sand tiger sharks [50]. Similarly, Gallagher et al. [13] found lactate to be the sole blood parameter to be significantly affected by increased time on the line for several shark species. The increase in lactate over time indicates that the “fight” on the line caused a shift to anaerobic respiration, which resulted in the accumulation of lactate as a byproduct. The elevated lactate levels (6.7 mmol/L on average) measured after 30 min in this study are consistent with previous research on other species, and similar increases in lactate are known to have negative physiological effects [43, 51]. Therefore, lactate values can serve as a proxy for assessing the immediate condition or health of a shark [43]. Fuller et al. [43] found lactate was able to significantly predict a shark’s behavior upon release with higher lactate values corresponding to more sluggish behavior post-release. Higher lactate values were also significantly correlated with moribund shortfin mako [52], blue [53], and blacktip sharks [44]. Although our study did not observe post-release behavior or record post-release mortality rates, the literature suggests the observed increases in lactate represent a considerable physiological cost that could impact short-term health, potentially rendering juveniles more susceptible to predation or disease.

Nurse sharks have regularly exhibited low behavioral [31, 32] and physiological response to capture stress. For example, Jerome et al. [14] found nurse sharks consistently had the lowest relative levels of physiological disturbance among studied species, including lactate levels and reflex impairment. Likewise, nurse sharks caught on longlines exhibited low-intensity exercise in response, with maximum lactate levels only reaching 3.25 mmol/L [54], compared to a maximum of 22.1 mmol/L in this study. However, previous studies reporting lower lactate results used commercial or scientific fishing methods (*e*.*g*., drumlines or longlines) where the nurse sharks are able to settle on the bottom for the duration of the soak time (1 hour). This is the first study to examine the effects of active recreational angling on nurse sharks, and the first to explore physiological stress in juvenile nurse sharks.

A period of hyperglycemia is expected after stress or exhaustive exercise in fish as utilization of glucose mobilized through the stress response or depletion of hepatic glycogen stores is suggested to occur [55–57]. Manire et al. [58] observed a substantial decrease in glucose for both bonnethead and bull sharks caught with gillnets. Conversely, significant increases in glucose levels were detected in both Atlantic sharpnose and blacktip sharks caught using hook-and-line [38, 44]. In this study, a weak relationship between glucose and fight time was measured. Results from the initial model showed a significant, positive relationship existed between glucose and total fight time for nurse sharks. However, in the final model, blood draw number was significant and fight time was nearly significant, which suggests juvenile nurse sharks may exhibit a more pronounced stress response from handling and air exposure than from short periods of exhaustive exercise. Longer periods of air exposure were found to increase blood-based disturbances and mortality rates in bonefish [59], and significantly disrupt physiological parameters in little skates (*Leucoraje erinacea*) with acute thermal stress exacerbating these impacts [60]. Previous work on elasmobranchs has also documented no meaningful change in glucose as a result of capture stress [50, 53, 61]. The combination of these results suggests changes in glucose values may be species-specific, a finding that is supported in the literature [52, 62]. It should be noted that the relationship and accuracy of the Accu-Chek glucose meter compared to values obtained with a colorimetric assay in the laboratory has not been validated for nurse sharks, due to insufficient laboratory data [41]. Inconsistencies between values obtained by the glucose meter versus a colorimetric assay could explain why there is only a weak relationship between glucose and fight time. Further investigation is needed to understand the accuracy of the Accu-Chek glucose meter.

Several previous studies have found that capture stress either causes significant increases in elasmobranch hematocrit values [57, 61, 63] or no significant changes [38, 53, 58]. Conversely, the final model for hematocrit in this study found blood draw number decreases hematocrit values. These lower hematocrit levels suggest that blood loss, potentially from the venipuncture site or hooking location, may result in hemodilution in small nurse sharks. This has been measured in other studies of elasmobranchs, and given that many studies engage in serial sampling including of small sharks [50, 61], should be considered in analysis. Repeated blood sampling of Port Jackson sharks and gummy sharks resulted in a significant decrease in hematocrit values, suggesting that the decline in hematocrit over the serial blood sampling protocol used here was likely a consequence of repeated blood extraction [62]. Similarly, Mohan et al. [44] measured significantly reduced hematocrit levels with increased handling times in blacktip sharks that were not related to the physiological stress associated with a capture event, and hypothesized the decrease was due to blood loss.

Capture stress can lead to disruption in the ability to osmoregulate in aquatic animals due to water shifting out of vascular compartments in response to increased intercellular lactate in the blood [38, 49, 64]. This is consistent with recent data showing an increase in osmolality with fight time in blacktip sharks [44] and Atlantic sharpnose sharks [42]. Interestingly, there was no evidence of a significant difference in osmolality in juvenile nurse sharks due to fight time. Similar results were observed in another study on blacktip sharks [43]. It is possible that the decrease in hematocrit, which in theory could also have resulted from plasma water moving into the circulation from the tissues, may have offset any increase in osmolality that occurred during the 30-min fight [49]. Additionally, it may take longer than 30 min for the effects of capture stress on osmolality to be clearly detectable.

It is well known that many shark nurseries are susceptible to environmental fluctuations in salinity due to their shallow, coastal nature [65]. In this study, osmolality varied significantly by fishing site with lower osmolality levels corresponding to sharks caught at a location near water outflows from canals that affect the salinity of surrounding habitats. Therefore, for species that typically have high site fidelity, local abiotic conditions may matter and proximity of the stress event to an outflow could affect the capture stress response. Additionally, coastal locations like the fishing sites in this study provide essential habitat for many elasmobranch species but are subjected to various anthropogenic disturbances. Aquatic animals are exposed to pollutants via the delicate respiratory surface of the gills and these environmental toxins are known to impair overall fish health and cause both acute and chronic stress [49]. Some studies have found that sharks residing in wastewater-impacted areas are exposed to and can accumulate measurable quantities of human pharmaceuticals [66]. Furthermore, Rangel et al. [67] found that juvenile nurse sharks sampled in highly impacted urban areas of Biscayne Bay in Miami had higher concentrations of plasma-saturated and bacterial fatty acids, suggesting they consumed lower-quality food resources than conspecifics in less urban-impacted areas. Therefore, these juvenile nurse sharks may already be under environmental stresses that could affect their long-term health and ability to respond physiologically to capture stress, which may pose a greater threat to these animals than previously believed. Additional research is needed to understand the relationship between environmental factors, especially in highly-human-impacted areas, and capture stress.

Although fishing effort for this study largely focused on the summer months, precluding concrete comparisons, the estimated Catch Per Unit Effort (CPUE) was approximately three times higher in this study in summer than in winter. This suggests juvenile nurse sharks may not be utilizing these popular fishing locations during the cooler months or may not be feeding as frequently, and therefore are likely caught less frequently during the winter months. In addition, model results revealed that higher lactate levels corresponded to warmer water temperatures; a relationship that has been previously noted for several other elasmobranch species [42, 68]. This indicates juvenile nurse sharks may experience increased capture stress during summer months when water temperatures are higher. Increased likelihood of being caught by recreational fishers and the amplified physiological response to capture during the summer months could represent a meaningful stressor to juvenile nurse sharks around South Florida, but this warrants additional research.

This study suggests juvenile nurse sharks exhibit a greater physiological stress response when exposed to recreational angling than some other shark species or their adult counterparts captured using other fishing methods. This provides insight into nurse shark stress responses that was not previously available, which can help to inform age- and practice-specific management and conservation strategies around shore-based recreational fisheries. Furthermore, results revealed blood draw number to be significant for lactate, glucose, and hematocrit, suggesting that handling and air exposure can cause substantial physiological stress for juvenile nurse sharks. These results and others suggest air exposure and handling by recreational anglers should be limited whenever possible, in line with other findings from recreational fisheries for teleosts [69, 70]. Therefore, policy decisions should be centered around reducing fight time as well as handling and air exposure in order to moderate the physiological stress experienced by juvenile nurse sharks caught on hook-and-line. Future studies should assess the primary endocrine stress response to capture as well as additional physiological variables (e.g., fatty acids and electrolytes) to gain a better understanding of the entirety of the capture stress response.

## Supporting information

S1 Dataset Complete dataset including fight times; total time a shark was on the line; hematocrit and osmolality values; hand-held point-of-care device lactate and glucose; shark measurements; shark sex; tag number; hook placement; release condition; capture location and GPS coordinates; water temperatures; tide times; and capture dates for each individual shark. (CSV)

S2 Dataset R script. Complete R script including both variations of linear mixed models (LMM) for all physiological parameters (the first for total fight time and the second for fight time that included blood draw number as a variable); the LMM for initial levels of each parameter and the time it took to obtain the initial blood sample; and the relative effect sizes for each model. (R)
